# *Emodin* induced necroptosis in the glioma cell line U251 via the TNF-α/RIP1/RIP3 pathway

**DOI:** 10.1007/s10637-019-00764-w

**Published:** 2019-03-28

**Authors:** Jiabin Zhou, Genhua Li, Guangkui Han, Song Feng, Yuhan Liu, Jun Chen, Chen Liu, Lei Zhao, Feng Jin

**Affiliations:** 1 Graduate School, Tianjin Medical University, Tianjin, 300070 People’s Republic of China; 2 Department of Neurosurgery, Affiliated Hospital of Jining Medical University, & Shandong Provincial Key Laboratory of Stem Cells and Neuro-oncology, Jining, Shandong 272029 People’s Republic of China; 3 Department of Traditional Chinese Medicine, Union Hospital, Tongji Medical College, Huazhong University of Science and Technology, Wuhan, 430022 People’s Republic of China; 4 Clinical Medical College, Jining Medical University, Jining, Shandong 272029 People’s Republic of China; 5 Department of Infectious Diseases, Union Hospital, Tongji Medical College, Huazhong University of Science and Technology, Wuhan, 430022 China

**Keywords:** Emodin, U251, Necroptosis, TNF-α, RIP1, RIP3

## Abstract

Emodin, an anthraquinone compound extracted from rhubarb and other traditional Chinese medicines, has been proven to have a wide range of pharmacological effects, such as anti-inflammatory, antiviral, and antitumor activities. Previous studies have confirmed that emodin has inhibitory effects on various solid tumors, such as osteosarcoma, liver cancer, prostate cancer and glioma. This study aimed to investigate the effects and mechanisms of emodin-induced necroptosis in the glioma cell line U251 by targeting the TNF-α/RIP1/RIP3 signaling pathway. We found that emodin could significantly inhibit U251 cell proliferation, and the viability of U251 cells treated with emodin was reduced in a dose- and time-dependent manner. Flow cytometry assays and Hoechst-PI staining assays showed that emodin induced apoptosis and necroptosis. Real-time PCR and western blot analysis showed that emodin upregulated the levels of TNF-α, RIP1, RIP3 and MLKL. Furthermore, the RIP1 inhibitor Nec-1 and the RIP3 inhibitor GSK872 attenuated the killing effect of emodin on U251 cells. In addition, emodin could increase the levels of TNF-α, RIP1, RIP3 and MLKL in vivo. The results demonstrate that emodin could induce necroptosis in glioma possibly through the activation of the TNF-α/RIP1/RIP3 axis. These studies provide novel insight into the induction of necroptosis by emodin and indicate that emodin might be a potential candidate for treating glioma through the necroptosis pathway.

## Introduction

Glioma is the most common primary malignant tumor in the central nervous system [1]. Because the etiology of glioma is uncertain, patients are not effectively treated. Moreover, although microneurosurgical techniques continue to be developed, tumor tissues cannot be completely resected as glioma cells are characterized by uncontrolled growth [2]. In addition, the development of radiotherapy and chemotherapy resistance in glioma is very common due to the existence of the subpopulation of cancer stem cells [3]. Thus, the mortality and recurrence rate of glioma are very high, and the median survival time of glioma patients is less than 16 months, even with standard treatment [1]. These characteristics show that apoptosis resistance and autophagy occurrence are important components of the development of resistance to malignant glioma therapy [4]. Therefore, it is urgent to find an effective new therapy to induce the death of glioma.

Emodin, one of the main effective components in traditional Chinese antitumor herbs [5], has been confirmed to have antitumor activity against many kinds of tumors, such as those in lung cancer [6], breast cancer [7], and colorectal cancer [8]. To date, most studies have demonstrated that emodin has the capability to accelerate apoptosis, induce autophagy, promote cell cycle arrest or inhibit tumor metastasis [9]. However, there is little research on emodin-induced necroptosis in glioma.

Necroptosis is one of the most important mechanisms of programmed cell death (PCD). The discovery of necroptosis provided a novel theoretical basis for tumor therapy [10] because necroptosis is independent from apoptosis and does not involve the activation of the caspase family [11]. In this study, we demonstrated that emodin simultaneously induced apoptosis and necroptosis. Moreover, the necroptosis induced by emodin was proven to be related to the activation of RIP1 and RIP3 in vitro and vivo.

## Results

### *Emodin* inhibited the viability of U251 cells but not LO2 cells

**Fig. 1:**
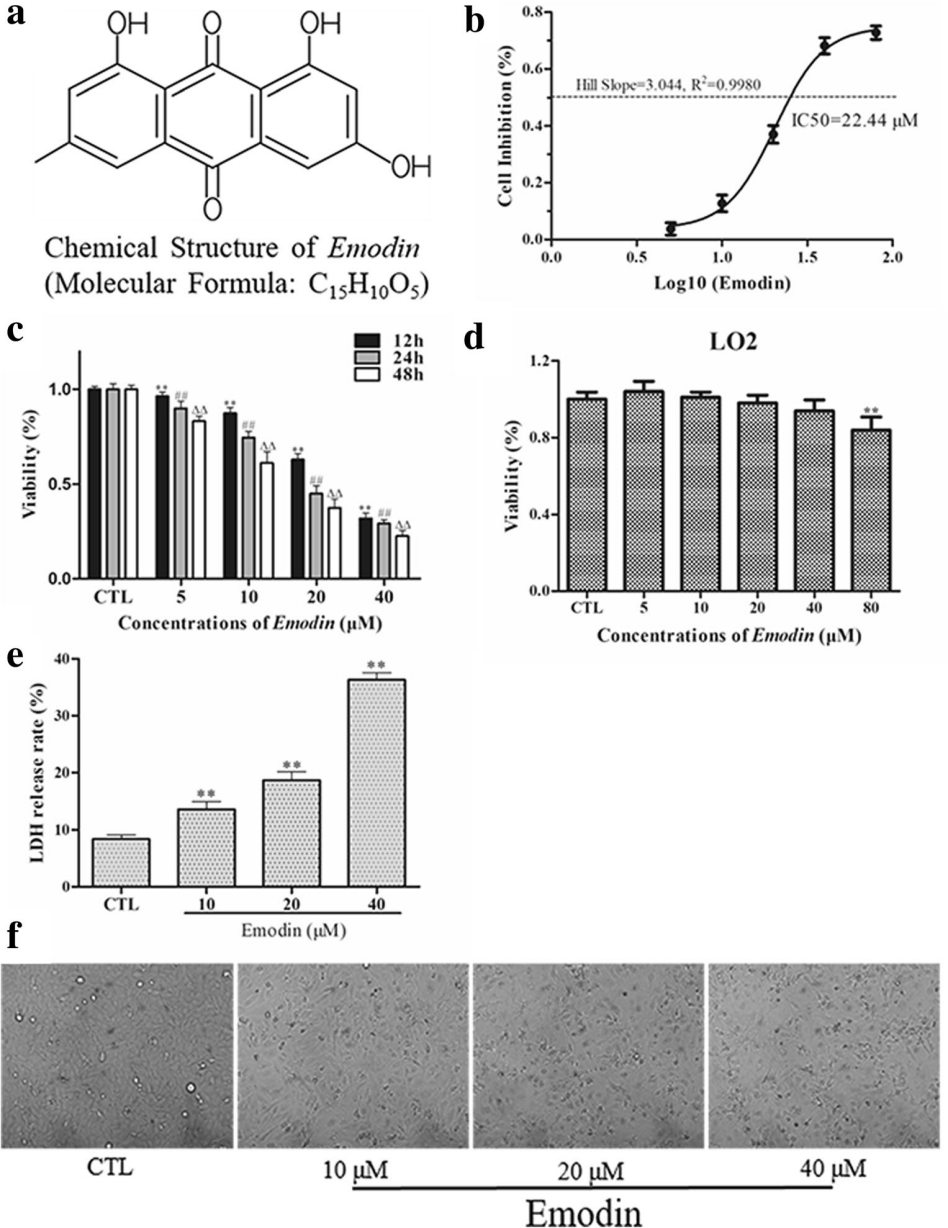
**a** The chemical structure of emodin. **b** The inhibitory effect of emodin on U251 cell proliferation as detected by CCK-8 assays after 12 h of treatment. **c** Dose- and time-dependent effects of emodin on U251 cell viability as determined by CCK-8 assays at 12, 24 and 48 h. ^**^P < 0.01, ^##^*P <* 0.01, and ^ΔΔ^*P <* 0.01 compared with the CTL group. (D) Cytotoxicity of emodin on LO2 cells as detected by CCK-8 assays after 12 h of treatment. **P < 0.01 compared with the CTL group. (E) LDH release test in U251 cells after 12 h of treatment with emodin. **P < 0.01 compared with the CTL group. (F) Morphology of U251 cells treated with different concentrations of emodin for 12 h

To confirm whether emodin could suppress the viability of U251 cells but not LO2 cells, a Cell Counting Kit-8 (CCK-8) assay was used to detect the viability of cells treated with different concentrations of emodin (Fig. 1b, c). The results showed that emodin could significantly attenuate the survival rate of U251 cells in a dose- and time-dependent manner. Specifically, we found that the half maximal inhibitory concentration (IC50) of emodin at 12 h was 22.44 μM (Fig. 1b). Therefore, we chose 10 μM emodin administered for 12 h as the lowest concentration. Additionally, emodin did not significantly change the viability of LO2 cells until the highest concentration was administered (Fig. 1d). Moreover, emodin dose-dependently increased the release of LDH from U251 cells (Fig. 1e).

### *Emodin* induced apoptosis, necrosis and cell cycle arrest in glioma U251 cells

**Fig. 2:**
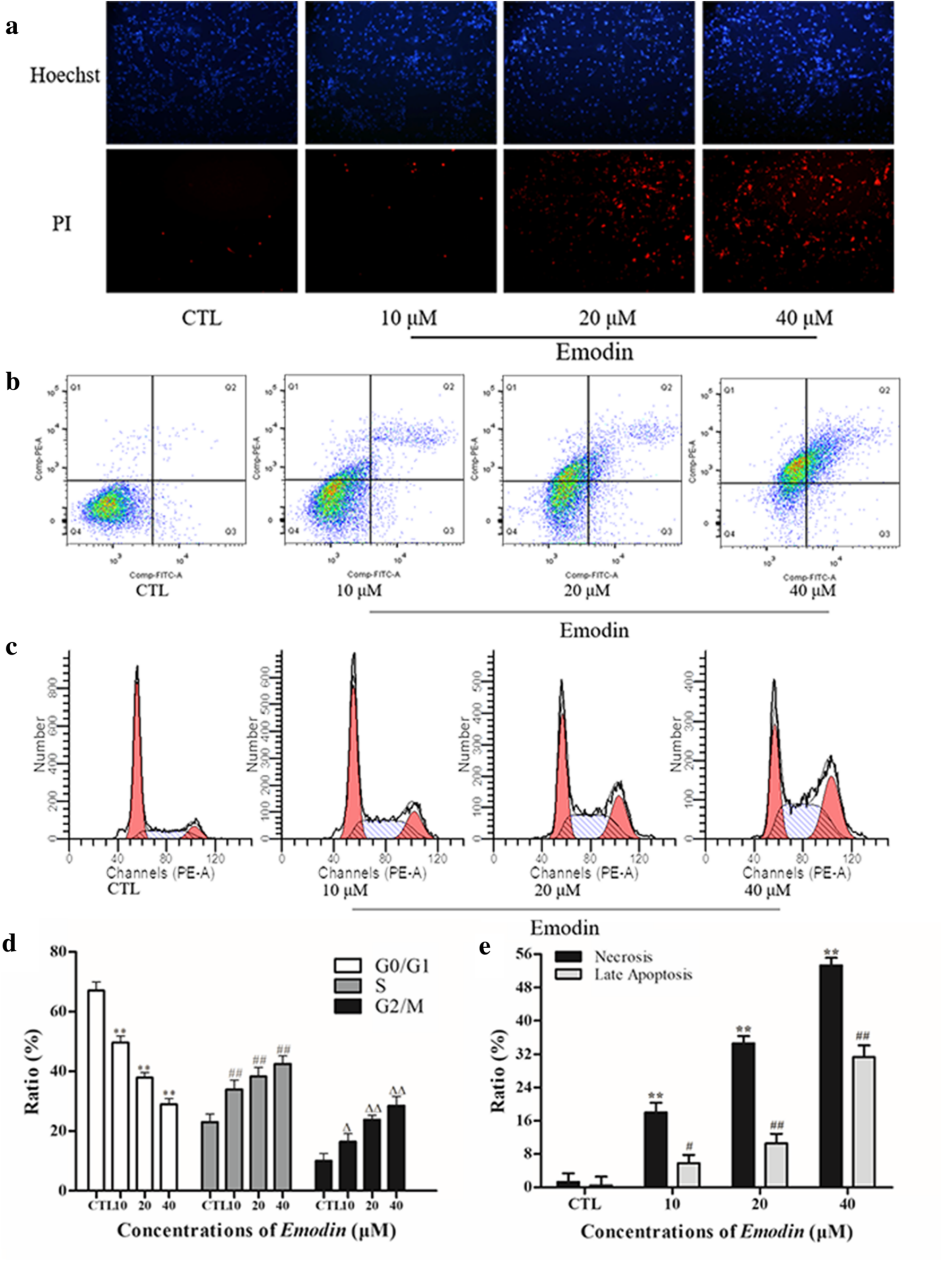
**a** Hoechst-PI double staining assay of U251 cells treated with emodin. **b**, **d** Emodin induced cell cycle arrest in U251 cells. Cells were treated with different concentrations of emodin for 12 h, and the samples were analyzed by flow cytometry. ^**^P < 0.01, ^##^*P <* 0.01, ^Δ^*P <* 0.05, and ^ΔΔ^*P <* 0.01 compared with the CTL group. **c**, **e** Different concentrations of emodin induce necrosis and apoptosis in U251 cells. Samples were analyzed by flow cytometry. ^**^P < 0.01, ^#^*P <* 0.05, and ^##^*P <* 0.01 compared with the CTL group

A Hoechst/propidium iodide (PI) double staining assay was used to detect cell morphology, apoptosis and necrosis. Fluorescent microscopy was used to observe U251 cells treated with emodin for 12 h. Normal U251 cells showed round nuclei with light blue color and no red color (Hoechst −/ PI -). Apoptotic U251 cells showed shrinking nuclei with dark blue color and no red color (Hoechst +/ PI -), while necrotic U251 cells showed shrinking nuclei with light blue color and red color (Hoechst −/ PI-). A flow cytometry assay with Annexin V-FITC and PI was used to detect apoptosis and necrosis. Emodin promoted the apoptosis and necrosis of U251 cells in a dose-dependent manner (Fig. 2c, e). The percentages of necrotic U251 cells treated with emodin for 12 h were 1.28 ± 2.08% (0 μM, control (CTL)), 18.0 ± 2.32% (10 μM), 34.6 ± 1.76% (20 μM), and 53.3 ± 1.83% (40 μM), while the percentages of late apoptotic U251 cells were 0.43 ± 2.11% (CTL), 5.81 ± 1.95% (10 μM), 10.5 ± 2.36% (20 μM), and 31.3 ± 2.86% (40 μM). Moreover, the ratio of U251 cells treated with emodin in the G0/G1 phase was decreased compared to CTL, but the ratios of cells in the S phase and G2/M phase were increased.

### *Emodin* not only promoted apoptosis by activating caspase-3 but also induced necroptosis in U251 cells via the TNF-α/RIP1/RIP3 pathway

**Fig. 3:**
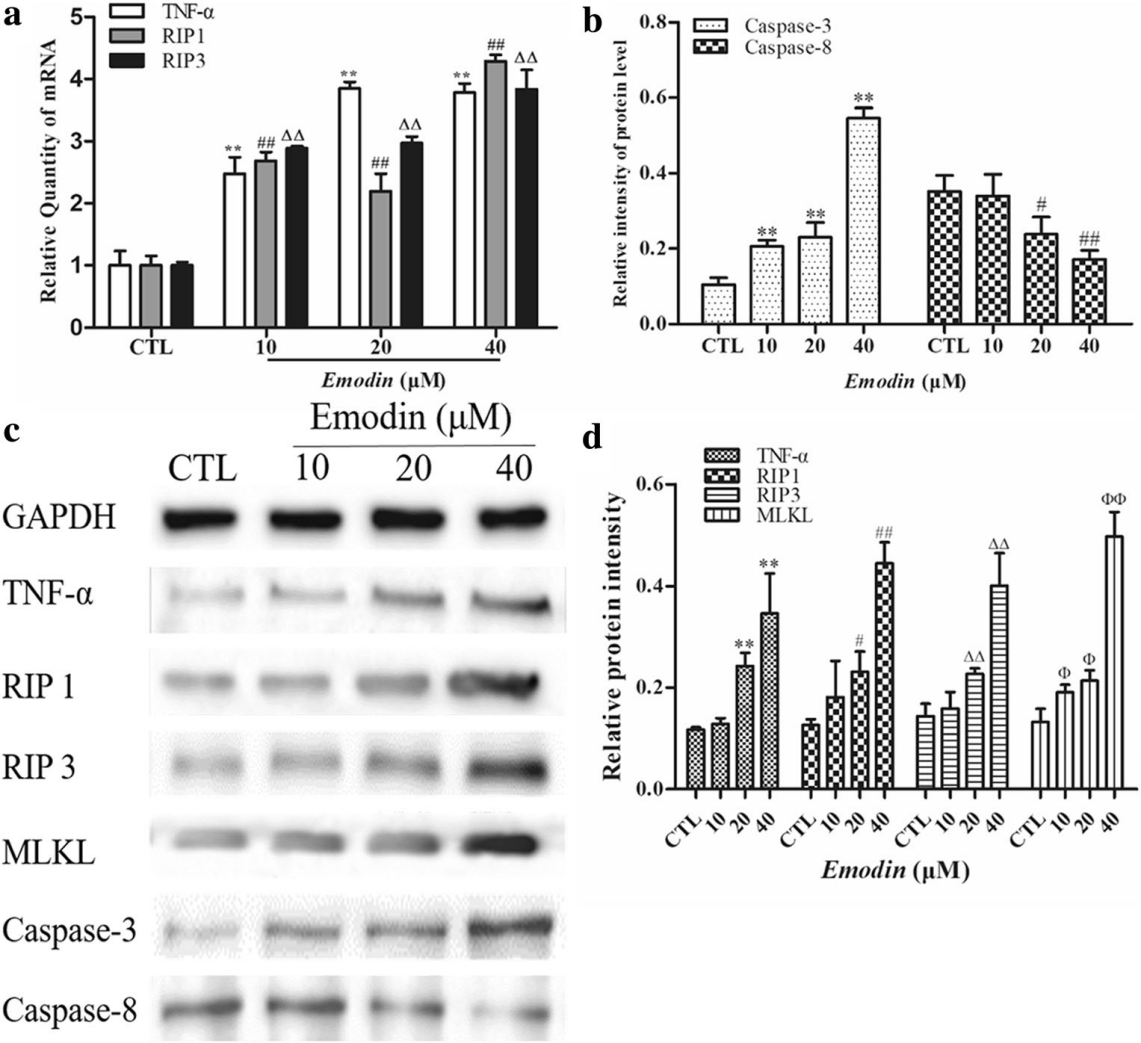
**a** RNA samples from U251 cells treated with emodin for 12 h were prepared and reverse-transcribed into cDNA. The mRNA levels of TNF-α, RIP1 and RIP3 were detected by a StepOne Plus device. Data were collected from three individual experiments. ^**^P < 0.01, ^##^*P <* 0.01, and ^ΔΔ^*P <* 0.01 compared with the CTL group. **b** The intensities of the caspase-3 and caspase-8 bands were quantified by the software ImageJ, and data were collected from three individual experiments. ^**^P < 0.01, ^#^*P <* 0.05, and ^##^*P <* 0.01 compared with the CTL group. **c** The protein of U251 cells treated with emodin for 12 h were prepared, and the levels of TNF-α, RIP1, RIP3, MLKL, caspase-3 and caspase-8 were measured by western blot analysis. **d**The intensities of the TNF-α, RIP1, RIP3 and MLKL bands were quantified by the software ImageJ, and data were collected from three individual experiments. ^**^P < 0.01, ^#^*P <* 0.05, ^##^*P <* 0.01, ^ΔΔ^*P <* 0.01, ^Φ^*P <* 0.05, and ^ΦΦ^*P <* 0.01 compared with the CTL group

The caspase family plays a key role in the cell death process, so we detected the protein levels of caspase-3 and caspase-8 in U251 cells treated with emodin. We found that the level of caspase-3 was increased with emodin treatment in a dose-dependent manner, but the level of caspase-8 was decreased (Fig. 3a, b). Combined with the above morphological results, we speculate that necroptosis may be the critical death mechanism induced by emodin in U251 cells. It is known that RIP1 and RIP3 are key regulators of necroptosis. Thus, we measured the mRNA and protein levels of TNF-α, RIP1, and RIP3 in U251 cells by real-time PCR and western blot analysis. We found that the mRNA and protein levels of all of these genes were increased with emodin treatment compared to CTL (Fig. 3a, c, d). Finally, these findings could preliminarily indicate that emodin induces necroptosis in U251 cells.

### The release of LDH caused by *emodin* in U251 cells could be attenuated by Nec-1 and GSK872

**Fig. 4:**
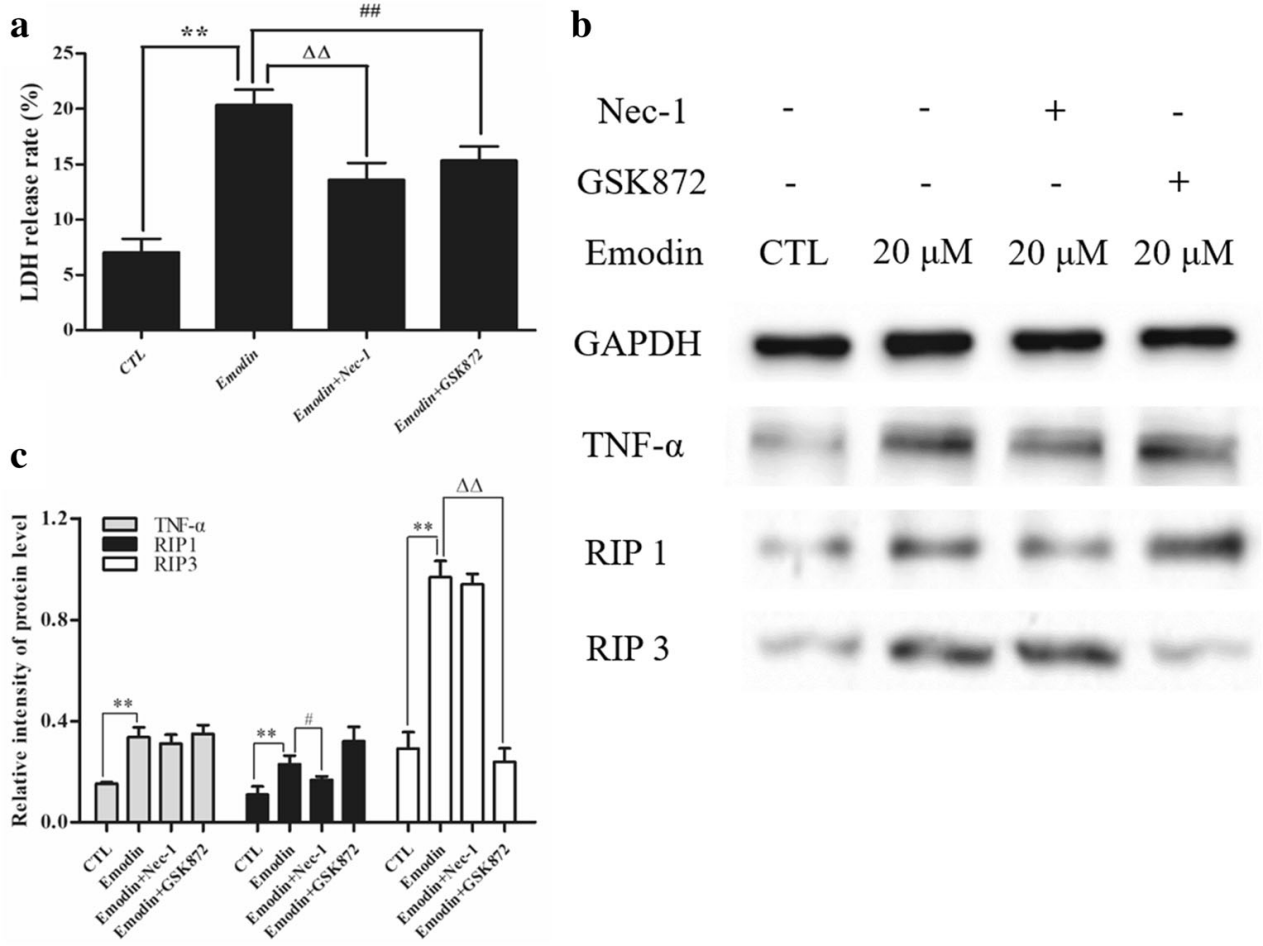
U251 cells were pretreated with Nec-1 and GSK872 for 2 h. **a** The emodin-induced release of LDH from U251 cells was determined by an LDH release assay. **P < 0.01, ##*P* < 0.01, ΔΔ*P* < 0.01. **b**, **c** Protein samples from U251 cells treated with emodin for 12 h were prepared, and the levels of TNF-α, RIP1 and RIP3 were measured by western blot analysis. The intensity of bands was quantified by ImageJ software, and data were collected from three individual experiments. ^**^P < 0.01, ^#^*P <* 0.05, ^Δ^*P <* 0.01

To demonstrate the role of RIP1 and RIP3 in the emodin-induced necroptosis of U251 cells, U251 cells were pretreated with the lowest concentration of necrostatin-1 (Nec-1) and GSk872 for 6 h, and then emodin was added for 12 h. LDH release assays showed that Nec-1 and GSK872 could significantly reduce the emodin-induced release of LDH from U251 cells (Fig. 4a). Moreover, western blot results suggested that Nec-1 could reduce the emodin-induced upregulation of RIP1, and GSK872 could inhibit the emodin-induced upregulation of RIP3; however, Nec-1 and GSK872 could not change the emodin-induced overexpression of TNF-α (Fig. 4b, c).

### *Emodin* inhibited glioma growth in vivo by regulating the TNF-α/RIP1/RIP3 pathway

**Fig. 5:**
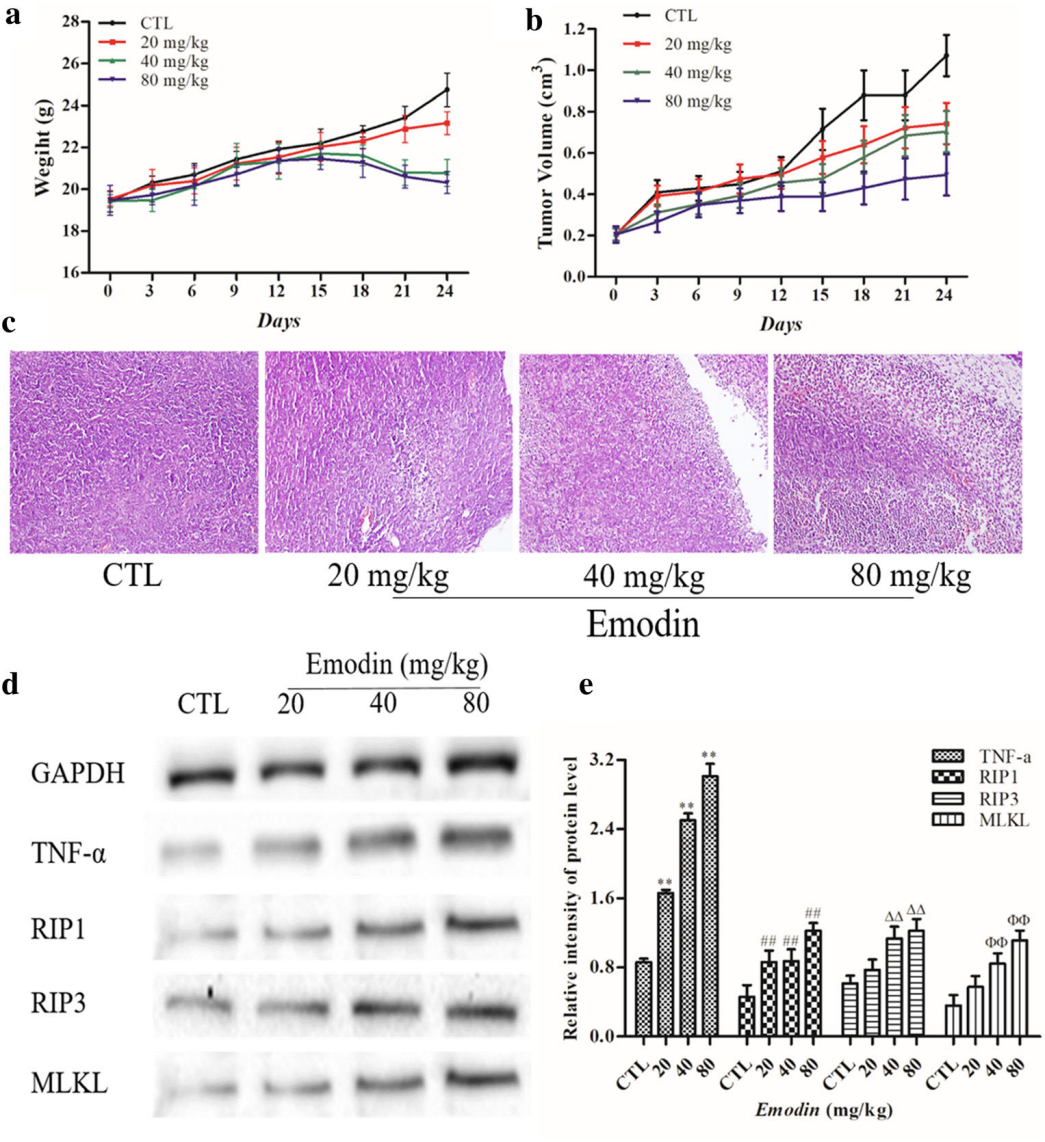
**a**, **b** Emodin decreases mouse weight and xenograft tumor volume. **c** H&E staining analysis of atypia and necroptosis in the control groups and emodin groups. **d**, **e** Protein samples from tumor tissues were prepared, and the levels of TNF-α, RIP1, RIP3 and MLKL were measured by western blot analysis. Data were collected from three individual experiments. ^**^P < 0.01, ^##^*P <* 0.01, ^ΔΔ^*P <* 0.01, and ^ΦΦ^*P <* 0.01 compared with the CTL group

To ascertain whether emodin exerted an antitumor effect on glioma through the TNF-α/RIP1/RIP3 pathway in vivo, we established a xenograft model by subcutaneously injecting U251 cells into BALB/C-nu/nu nude mice [12]. The nude mice were randomly assigned to four groups (six mice per group). Solid tumors were initially formed for almost two weeks, and the tumor formation rate was 100%. Then, tumor-bearing nude mice were treated with emodin (20, 40, and 80 mg/kg) or the same volume of saline (negative CTL) by intragastric administration for four weeks, and there were no dead mice until execution. As shown in Fig. 5a, b, emodin could inhibit the growth of tumors, and the mean volume and weight of tumors in the emodin groups were lower than those in the CTL group. Hematoxylin-eosin (H&E) staining showed that there were obvious atypia nuclei, poor differentiation, and a small amount of necrosis in the tumor tissues in the CTL group; however, there was massive necrosis in the emodin-treated group (Fig. 5c). To investigate whether emodin induced necroptosis in vivo, we measured the levels of TNF-α, RIP1, RIP3 and MLKL in tumor tissues treated with emodin or saline. The results showed that compared with the CTL group, the levels of TNF-α, RIP1, RIP3 and MLKL were increased significantly in the emodin-treated group (Fig. 5d, e). These results suggested that emodin could suppress glioma growth through the necroptosis pathway in vivo.

## Discussion

Previous studies considered cell death to be divided into PCD and necrosis. PCD, which is also called apoptosis, is distinct from necrosis and is a death process initiated by specific genes. The occurrence of apoptosis is dependent on the activation of the caspase family and apoptosis-related genes, and the promotion of the protein degradation pathway makes the apoptosis of cells irreversible [13]. The most typical morphological characteristics of apoptotic cells are karyopyknosis, nuclear fragmentation and apoptotic body formation, but this process is not related to cell membrane changes; the membrane of apoptotic cells is continuous. In contrast, the permeability of necroptotic cells is increased, and the cellular membrane is severely destroyed. In addition, necroptosis is always coupled with high levels of LDH release. In our study, we preliminarily confirmed that the inhibition of U251 cells induced by emodin was associated with necroptosis.

Previous studies showed that necrosis is a type of passive death; necrosis is unavoidable under harmful stimulation. However, accumulating evidence has confirmed that necrosis is also regulated by some necrotic-associated genes but is not dependent on the caspase signaling pathway. Necrosis is expressed in many forms, including necroptosis [14], ferroptosis [15], and pyroptosis [16]. Necroptosis is one of the most well studied forms of necrosis; it is induced by the activation of the death receptor on the cell membrane and is dependent on the formation of the RIP1/RIP3/MLKL complex (called necrosomes) [17]. Therefore, RIP1, RIP3 and MLKL are the core molecules in the necroptosis signaling pathway [18].

Necroptosis has been well studied in the fields of inflammatory response and ischemia-reperfusion injury [18], but there has been less research on necrotic tumor therapy. For a long time, studies examining antineoplastic agents have focused on their pro-apoptosis effects, but drug resistance has resulted in poor therapeutic effects [19]. Apoptosis dysfunction is a core factor in the resistance of almost all tumors, especially in glioma, to radio-chemotherapy [20]. As it does not depend on caspase activation, the induction of necroptosis was an eximious therapeutic strategy for tumor treatment, particularly for drug-resistant tumors. Notably, many chemotherapeutic drugs and molecular-targeted agents that induce necroptosis have been used in clinical trials.

Emodin, also called 3-methyl-1,6,8-trihydroxyanthraquinone, is an important active component extracted from traditional Chinese herbs [21]. Emodin has been reported to have various pharmacological activities, such as anti-inflammatory [11], antibiotic, antihypertensive and antitumor activities; however, studies examining the antitumor effects of emodin have almost all focused on apoptosis induction [22], invasion and migration inhibition [23] as well as cell cycle arrest. Nevertheless, no specific studies have reported the effects and mechanisms of necroptosis induced by emodin on glioma cells. Our study found that emodin could induce apoptosis by activating caspase-3; however, surprisingly, the protein levels of caspase-8 [24] were decreased in glioma U251 cells treated with emodin.

TNF-mediated necroptosis is the most commonly studied type of necroptosis cell death [25]. As an initiator of necroptosis, TNF-α receptors bind with ligands on the membrane, downstream molecules are activated, and the necroptosis pathway is promoted [26], including the formation of membrane-associated complex I, complex II and necrosomes (inhibition of caspase-8 and phosphorylation of RIP1/RIP3/MLKL) [27, 28]. In our studies, we found that the both the mRNA and protein levels of TNF-α, RIP1, RIP3 and MLKL were increased in glioma cells treated with emodin in vivo and in vitro for the first time. Therefore, we conjectured that emodin could induce necroptosis by activating the expression of TNF-α and downstream molecules. As previously reported, Nec-1 [29], an inhibitor of RIP1, and GSK872 [29], an inhibitor of RIP3, could inhibit necroptosis by inhibiting RIP1 and RIP3, respectively [30]. To evaluate our hypothesis, U251 cells treated with emodin were pretreated with Nec-1 (inhibitor of RIP1) and GSK872 (inhibitor of RIP3). Subsequently, we found that the decrease in the viability of U251 cells treated with emodin could be attenuated by Nec-1 and GSK872. In our studies, emodin promoted the necroptosis of U251 cells by activating RIP1 and RIP3.

Unfortunately, we could not prove the role of TNF-α in the process of emodin-induced necroptosis. Because of the complicated biological activities of emodin, it may induce necroptosis by other pathways. For future studies, researchers should consider the combined application of antitumor agents by inducing apoptosis, necroptosis and other ways of cell death [31]. Additionally, it is necessary to pay more attention to the side effects and potential risk of emodin in normal individuals. Taken together, these studies provide valuable insights into the molecular mechanisms of emodin, and our findings may facilitate studies of emodin as a potential candidate for the treatment of gliomas and other tumors.

## Materials and methods

### Chemicals and reagents

Dulbecco’s modified Eagle’s medium/nutrient mixture F12 (DMEM/F12), fetal bovine serum (FBS), and dimethyl sulfoxide (DMSO) were purchased from GIBCO (Grand Island, NY, USA). Emodin (CAS No. 518–82-1, HPLC>98%) was obtained from Dalian Meilun Biotechnology Co. Ltd. (Dalian, China). RNAiso plus, SYBR Premix Ex Taq II and ROX reference dye were purchased from TAKARA (Dalian, China). All primers were designed and synthesized by TSINGKE (Wuhan, China). The following antibodies for western blot analysis were purchased from ABclonal Biotechnology Co., Ltd. (Wuhan, China): GAPDH; caspase-3; caspase-8; TNF-α; RIP1; RIP3; MLKL; and HRP-labeled goat anti-rabbit IgG. Emodin was dissolved in DMSO as a stock solution and stored in a dark bottle at 4 °C. Nec-1 (CAS No.: 4311-88-0) and GSK872 (CAS No.:1346546–69-7) were purchased from MedChemExpress (Shanghai, China). The FITC Annexin V Apoptosis Detection Kit I (556547) was purchased from BD (USA). CCK-8 was purchased from Dojindo (Japan). The Apoptosis and Necrosis Assay Kit, Cell Cycle and Apoptosis Kit, and LDH Assay Kit were all purchased from Beyotime (China).

### Cell culture

The human glioma cell line U251 was purchased from the China Center for Type Culture Collection (CCTCC). The human embryo liver cell line LO2 was characterized by Professor Lei Zhao (Union Hospital, Tongji Medical College, Huazhong University of Science and Technology). The LO2 and U251 cells were cultured in DMEM/F12 supplemented with 10% FBS and 1% penicillin-streptomycin in a 5% CO_2_ humidified incubator at 37 °C.

### Cell viability assay

LO2 and U251 cells were plated at a density of 4 × 10^3^ cells per well into 96-well plates and incubated overnight. Then, different concentrations of emodin were added for 12, 24 and 48 h. A total of 10 μL of CCK-8 solution was added to each well and incubated for 2 to 4 h. The absorbance was measured at 450 nm by using a spectrophotometer.

### Observation of cell morphology

U251 cells were seeded in 6-well plates and incubated overnight. The next morning, cells were treated with different concentrations of emodin for 12 h. Then, cell morphology was observed and photographed by an inverted microscope (Olympus, Japan).

### Apoptosis and cell cycle analysis by flow cytometry

U251 cells were treated with different concentrations of emodin for 12 h. Cells were collected and washed three times with phosphate-buffered saline (PBS). For the detection of apoptosis, cells were stained with the reagents in the FITC Annexin V Apoptosis Detection Kit according to the instructions. For the detection of the cell cycle, cells were stained with the reagents in the Cell Cycle and Apoptosis Kit according to the instructions. All samples were analyzed by flow cytometry (BD, USA).

### Apoptosis and necroptosis analysis

U251 cells were plated at a suitable density into 12-well plates and incubated overnight. Cells were treated with different concentrations of emodin for 12 h. Cells were collected and processed according to the instructions of the Apoptosis and Necrosis Assay Kit. After incubation for 30 min protected from light, the cells were observed and photographed by an inverted fluorescence microscope (Olympus, Japan).

### LDH release assay

U251 cells were seeded into 96-well plates and cultured overnight. Different concentrations of emodin were added to the plates for 12 h, and then the samples were processed according to a portion of the LDH release assay instructions. The concentrations of LDH in the medium were measured by a spectrophotometer at a wavelength of 490 nm.

### RNA isolation and real-time quantitative PCR (qRT-PCR)

Total RNA was extracted from cells and tumor tissues by using RNAiso. cDNAs were reverse-transcribed from quantified RNA samples by using a PrimeScript RT Reagent kit at 37 °C for 15 min and 85 °C for 5 s. Then, the SYBR Premix Ex Taq Kit was added to the cDNAs according to the instructions. A StepOne Plus device (Applied Biosystems) was used to perform real-time PCR at 95 °C for 10 s followed by 40 cycles of 95 °C for 5 s and 60 °C for 20 s. The primers used are provided in Table 1.

**Table 1:** Sequences of Primers for RT-PCR

Genes	Primers
TNF-α	Forward: CCTCTCTCTAATCAGCCCTCTG
	Reverse: GAGGACCTGGGAGTAGATGAG
RIP1	Forward: TTACATGGAAAAGGCGTGATACA
	Reverse: AGGTCTGCGATCTTAATGTGGA
RIP3	Forward: CATAGGAAGTGGGGCTACGAT
	Reverse: AATTCGTTATCCAGACTTGCCAT
MLKL	Forward: AGGAGGCTAATGGGGAGATAGA
	Reverse: TGGCTTGCTGTTAGAAACCTG
β-actin	Forward: CATGTACGTTGCTATCCAGGC
	Reverse: CTCCTTAATGTCACGCACGAT

### Western blot analysis

Total protein was extracted and quantified from cells and tumor tissues by using RIPA [50 mM Tris (pH 7.4), 150 mM NaCl, 1% Triton X-100, 1% sodium deoxycholate, 0.1% SDS, sodium orthovanadate, sodium fluoride, EDTA, leupeptin] with additional PMSF. Proteins were loaded into wells, separated on 10% SDS-PAGE gels, transferred to PVDF membranes, saturated and blocked with 5% fat-free milk for 1 h. Then, the membranes were probed with primary antibodies and secondary antibodies. The signals were detected by a UVP BioSpectrum Imaging System after the membranes were soaked in enhanced ECL reagents. The intensity of all signals was quantified by ImageJ software.

### Xenograft models

Twenty-four BALB/C nude mice (female, 35–41 days, weighing 18–21 g) were purchased from Beijing Vital River Laboratory Animal Technology Co., Ltd. (Beijing China). U251 cells (5 × 10^6^ cells/200 μL) were subcutaneously injected into the right hindleg of the mice. After almost 7 days, the size of tumors was approximately 100 mm^3^. The 24 tumor-bearing mice were equally divided into four groups: high dose of emodin (80 mg/kg); middle dose of emodin (40 mg/kg); low dose of emodin (20 mg/kg); and CTL. Then, 0, 20, 40, and 80 mg/kg of emodin were given to each group by intragastric administration every day. After four weeks, the mice were sacrificed, and the tumors were collected.

### Hematoxylin & eosin (H&E) staining assay

Tumor tissues were fixed in 4% formaldehyde, embedded in paraffin and cut into 4 mm thick sections. The sections were deparaffinized in dimethylbenzene for 5 min and put into alcohol for 3 min. Sections were washed with distilled water and soaked in hematoxylin staining solution for 15 min. Next, the sections were washed with distilled water to remove the excess staining solution and placed in 0.5–1% hydrochloride alcohol for 10 s. Then, the sections were washed with running water for 15 min and stained with eosin (0.1–0.5%) for 5 min. Finally, the sections were hyalinized with dimethylbenzene for 10 min and dehydrated with alcohol for 3 min. The sections were dropped by neutral gum and covered by slides. The sections were observed and photographed by inverted microscopy. In the resulting images, the nucleus was blue, while the cytoplasm and the extracellular matrix were red.

### Statistical analysis

All statistical data were analyzed by using GraphPad Prism software v5.0, and grayscale images were obtained by ImageJ software. Data are expressed as the mean ± SD of three independent experiments. The significance of the statistical results was determined by *one-way* ANOVA with the post hoc *Dunnett’s test*. *P < 0.05* was considered statistically significant.
